# 

**Published:** 1894-04

**Authors:** 


### CONTENTS.

ORIGINAL ARTICLES—

Epilepsy. By J. A. Larrabee, M. D., Louisivlle, Ky.......................... 175
Pneumonia. By Thomas Turnbull, Jr., M.D., Hartford, Conn........... 183

SOCIETY REPORTS—

Atlanta Society of Medicine.................................................. 190
Clinical Society of Maryland.................................................. 192
Richmond Academy of Medicine and Surgery ................................ 198

SELECTIONS AND ABSTRACTS—

On the Restriction and Prevention of Tuberculosis........................ 202
Rest in Cardiac Affections ...................................................... 204
A Mode of Compressing the Abdominal Aorta............................ 205
Points for Trained Nurses........................................................ 206
A New and Quick Furniture Polish.......................................... 206
Operative Interfernce During the Second Stage of Labor.................. 207
A New Treatment for Dysentery................................................ 208
Irrigation of the Stomach in the Treatment of Incoercible Hiccough 210
The Treatment of Eclampsia.................................................... 211
Practical Points in the Treatment of Hip Disease. ........................ 212

SELECTIONS AND ABSTRACTS—CONTINUED—

Resection of the Inferior Maxillary Nerve .......... 213
To Raise the Standard of Medical Education .......... 214
"Doc" .......... 215

EDITORIAL—

Medical Association of Georgia .......... 216
Fifth Commencement of the Chattanooga Medical College .......... 219
Sixteenth Commencement of the Southern Medical College .......... 219

EDITORIAL NOTES .......... 221

PRESCRIPTION DEPARTMENT .......... 224, 225

Granular Conjunctivitis—Epistaxis—Diabetes Insipidus—Night-sweats of Phthisis—Mental Excitement of Hysteria—Oxalic Acid as an Emmenagogue—Prophylactic Against Frequently Recurring Tonsillitis—Nasal Catarrh—Dysentery in Children—Insomnia of Children.

SPECIAL NOTES .......... 226

Sanders & Son's Eucalypton Extract—Sanmetto Does not Simply Obscure Pathology, but it Cures.

